# Diagnostic challenges of diabetic kidney disease

**DOI:** 10.11613/BM.2023.030501

**Published:** 2023-08-05

**Authors:** Marijana Vučić Lovrenčić, Sandra Božičević, Lea Smirčić Duvnjak

**Affiliations:** 1 Department of clinical chemistry and laboratory medicine, University hospital Merkur, Zagreb, Croatia; 2 Vuk Vrhovac University clinic for diabetes, endocrinology and metabolic diseases, University hospital Merkur, Zagreb, Croatia; 3 School of medicine, University of Zagreb, Zagreb, Croatia

**Keywords:** diabetic nephropathy, glomerular filtration rate, albuminuria, biomarkers, diagnostic tests

## Abstract

Diabetic kidney disease (DKD) is one of the most common microvascular complications of both type 1 and type 2 diabetes and the most common cause of the end-stage renal disease (ESRD). It has been evidenced that targeted interventions at an early stage of DKD can efficiently prevent or delay the progression of kidney failure and improve patient outcomes. Therefore, regular screening for DKD has become one of the fundamental principles of diabetes care. Long-established biomarkers such as serum-creatinine-based estimates of glomerular filtration rate and albuminuria are currently the cornerstone of diagnosis and risk stratification in routine clinical practice. However, their immanent biological limitations and analytical variations may influence the clinical interpretation of the results. Recently proposed new predictive equations without the variable of race, together with the evidence on better accuracy of combined serum creatinine and cystatin C equations, and both race- and sex-free cystatin C-based equation, have enabled an improvement in the detection of DKD, but also require the harmonization of the recommended laboratory tests, wider availability of cystatin C testing and specific approach in various populations. Considering the complex pathophysiology of DKD, particularly in type 2 diabetes, a panel of biomarkers is needed to classify patients in terms of the rate of disease progression and/or response to specific interventions. With a personalized approach to diagnosis and treatment, in the future, it will be possible to respond to DKD better and enable improved outcomes for numerous patients worldwide.

## Introduction

Diabetes mellitus is a chronic progressive illness with an ever-increasing global prevalence, which is regarded as a consequence of the obesity epidemic and a sedentary lifestyle (*1*, *2*). Numerous devastating complications of diabetes significantly affect morbidity and mortality and provide an immense clinical and socio-economic burden across the world (*3*, *4*).

Diabetic kidney disease (DKD) is one of the most common microvascular complications of both type 1 and type 2 diabetes and the most common cause of the end-stage renal disease (ESRD) (*5*-*7*). Patients with DKD have a reduced quality of life and an increased risk of adverse outcomes such as ESRD, cardiovascular events and increased mortality (*8*, *9*).

About 50% of all patients with type 2 diabetes (T2DM) and 30% of those with type 1 diabetes (T1DM) will develop chronic kidney disease (CKD) (*6*, *10*). However, it is difficult to determine the exact share of diabetes in the development of CKD, because other factors that contribute to kidney dysfunction are often present, especially in patients with type 2 diabetes (*11*). These factors range from hypertension and obesity to glomerular atherosclerosis and age-associated loss of kidney function (*10*). Therefore, a more accurate term would be chronic kidney disease in diabetes, rather than diabetic kidney disease.

Diabetic kidney disease is clinically defined by decreased glomerular filtration rate (GFR) or elevated urinary albumin excretion (UAE), or both (*12*). Diabetic kidney disease was originally defined by Mogensen 40 years ago as a progressive kidney disease in diabetic patients, beginning with the loss of small amounts of albumin in the urine (30-300 mg *per* day) (*13*). This stage was termed microalbuminuria or incipient nephropathy. Over time, the progressive increase in albuminuria reaches the sensitivity cut-off for protein detection with the common urinary dipstick (> 300 mg/day), and macroalbuminuria or overt nephropathy occurs. This is followed by the decline in renal function, progressive kidney damage, and finally ESRD. Microalbuminuria has long been identified as a sensitive marker of the glomerular basement membrane damage, which is one of the early stages in the pathogenesis of DKD. However, a decline in estimated GFR (eGFR) is observed in a significant proportion of diabetic patients with normal albuminuria, indicating complex and diverse pathways involved in the development of DKD, including not only glomerular, but also tubular and extra-renal targets (*11*, *14*).

## Pathophysiology

The pathophysiology of DKD is a complex process involving the interplay of genetic and environmental factors that trigger pathogenetic mechanisms responsible for kidney damage (*15*). Both genome-wide association and single-gene polymorphism studies have pinpointed genetic variants of the genes coding for various metabolic pathways, but the results are hardly reproducible and the genetic map of DKD remains unclear. As evidenced in a recent meta-analysis, 66 genetic polymorphisms and six signalling pathways ranging from inflammation and endothelial dysfunction to lipid and glucose metabolism, as well as the renin-angiotensin-aldosterone system (RAAS) were associated with DKD (*16*). Among others, significant associations with DKD were detected for angiotensin-I-converting enzyme, carnosine dipeptidase 1, methylenetetrahydrofolate reductase, nitric oxide synthase 3, interleukin 1B and sirtuin 1 gene polymorphisms (*16*).

Epigenetic mechanisms and metabolic memory-associated expression of DKD-related genes in the hyperglycaemic *milieu* have also been implicated and intensively studied in DKD pathogenesis (*17*). Nevertheless, the majority of metabolic and haemodynamic disturbances that underlie specific structural and functional abnormalities are primarily caused by hyperglycaemia (*18*).

Diabetic kidney disease is a renal manifestation of the ubiquitous pathogenetic process driven by hyperglycaemia targeting vascular endothelial cells. Diabetic retinopathy, neuropathy and DKD cluster into microvascular complications, whereas coronary artery disease, peripheral vascular and cerebrovascular disease result from the same pathogenetic process targeting large blood vessels endothelial lining and therefore termed as macrovascular complications (*10*). Thickening of the glomerular basement membrane is the main characteristic of diabetes. It occurs as a consequence of the dysfunction of both podocytes and endothelial cells, caused by the toxic effect of hyperglycaemia, whereby inflammatory mediators and reactive oxygen species (ROS) play a significant role. Thickening of the glomerular basement membrane is responsible for early functional hyperfiltration and albuminuria, while disturbed haemodynamic processes further aggravate the glomerular damage (*10*). Scientific evidence supports the role of deranged sodium-glucose cotransporter 2 (SGLT2) and the RAAS as upstream mechanisms operative in the early hyperfiltration stage (*11*, *19*). Another characteristic change in DKD is the proliferation and hypertrophy of mesangial cells, which additionally contribute to glomerulopathy *via* increased production and accumulation of matrix proteins affecting both glomerular structure and function (*20*). Furthermore, in response to mesangial expansion and tissue injury, infiltration and activation of inflammatory cells additionally contribute to kidney damage by means of known biochemical mediators such as cytokines and ROS (*21*).

Apart from the glomerular basement membrane, proximal tubules are also damaged by hyperglycaemia, operating primarily *via* a high glucose transport state and associated local hypoxia (*22*). In the initial phase of DKD, there is a maladaptive hypertrophy and hyperplasia of tubules, followed by the progressive and cumulative atrophy of tubular epithelial cells resulting in alterations in tubuloglomerular feedback, and tubulointerstitial inflammation and fibrosis eventually. Mechanisms playing a significant role in tubular damage are again a vicious circle of inflammation and oxidative damage, together with mitochondrial dysfunction and endoplasmic reticulum (ER) stress (*15*, *22*, *23*). An emerging role of lipotoxicity in the pathogenesis of DKD is supported by the evidence highlighting lipotoxic and lipoapoptotic pathways in both the podocytes and proximal tubule cells, as well as genetic studies identifying aberrations in lipid metabolic signalling pathways (*16*, *24*).

## Diagnostic approach

Diabetic kidney disease is a slow-onset complication of diabetes developing over time. In patients with type 1 diabetes, it usually develops some 10 years after diagnosis, while in type 2 diabetes CKD it is not unusual to be present at the time of diagnosis, since type 2 diabetes often remains undiagnosed until after the complications have become apparent. It has been evidenced that targeted interventions at an early stage of DKD can efficiently prevent or delay the progression of kidney failure and improve patient outcomes (*25*). Therefore, regular screening for DKD has become one of the fundamental principles of diabetes care. Current clinical guidelines recommend at least annual screening for DKD in patients with type 1 diabetes lasting more than 5 years, in all patients with type 2 diabetes, and in all patients with hypertension and diabetes (*26*). Once detected, DKD is treated according to clinical guidelines to optimize glycaemic and blood pressure control and followed up at regular intervals (*26*). Clinical diagnosis of DKD is defined as the presence of albuminuria in at least two, out of three urinary samples collected within 3-6 months, and/or reduced GFR, if other causes of kidney damage were excluded. A non-diabetic CKD should be suspected if there is a fast decline in GFR, active urinary sediment, refractory hypertension or nephrotic-range albuminuria in a patient with diabetes (*27*).

Two simple laboratory tests are used to detect and classify the stage of CKD in diabetes: UAE and serum creatinine-based estimated GFR (SCr-eGFR), as calculated with a validated CKD-EPI formula (*28*). Well-established clinical prognostic stages based on the test results are used to classify the degree of kidney damage (Table 1) (*29*). There are three stages of albuminuria: normal to mildly increased (A1), moderately increased (A2) and severely increased (A3). Estimated GFR is staged into six categories: normal to high (G1), mildly decreased (G2), mildly to moderately decreased (G3a), moderately to severely decreased (G3b), severely decreased (G4) and kidney failure (G5) (Table 1).

**Table 1:** Thresholds of CKD classification according to eGFR and albuminuria

**eGFR**				**Albuminuria**	
**Stage**	**eGFR** **(mL/min/1.72 m^2^)**	**Stage**	**UACR (mg/g)**	**UACR (mg/mmol)**	**UAER (mg/24h)**
G1	≥ 90	A1	< 30	< 3	< 30
G2	60-89				
G3a	45-59	A2	30-300	3-30	30-300
G3b	30-44				
G4	15-29	A3	> 300	> 30	> 300
G5	< 15				
CKD - chronic kidney disease. eGFR - estimated glomerular filtration rate. UACR - urinary albumin to creatinine ratio. UAER - urinary albumin excretion rate. Adapted from reference 30.					

Since the decline in GFR is a part of normal ageing, a universal eGFR cut-off for CKD set at 60 mL/min/1.73m^2^ has been recently challenged, and age-specific thresholds proposed to improve screening for CKD (*30*).

However, despite widely accepted long-term clinical use and standardization efforts, laboratory tests for the screening and classification of DKD still suffer from many of analytical and biological confounders, and the equations used for the GFR estimates have limitations in specific patient populations. Besides, given an evolving knowledge of the complex pathophysiological mechanisms and genetic and epigenetic factors that translate into diverse clinical phenotypes of the CKD in diabetes, a more personalized approach with specific biomarkers is needed to achieve appropriate diagnostic tools not only for the early detection of kidney damage in diabetes but also for the targeting of the most efficient interventions (*12*). Diagnostic challenges of the recommended tests, as well as emerging biomarkers for DKD, are discussed in the following sections.

## Serum creatinine

Serum creatinine concentration has been used for many years as a marker of glomerular filtration for practical reasons, despite serious biological and analytical limitations (*31*). Namely, creatinine concentrations are significantly influenced by eating habits, muscle mass, age, gender and ethnicity. In addition, creatinine is not a specific marker of glomerular filtration due to its partial excretion and reabsorption in the tubules (*32*).

Despite the completed standardization process, the clinical reliability of creatinine is still burdened by some unresolved analytical issues (*33*). Still frequently used modified Jaffe alkaline picrate method suffers from the interference of non-specific chromogens such as glucose, proteins, and other reducing compounds, which may affect the accuracy of GFR estimation in patients with pronounced hyperglycaemia (*34*, *35*). In addition, an improvement of the accuracy in the lower concentration range is urgently needed for patient populations with low muscular mass, primarily paediatric (*36*). An universal use of enzymatic creatinine tests has been advocated, due to superior specificity, lower imprecision and bias compared to the reference method-isotope dilution/mass spectrometry (ID/MS) (*37*). However, enzymatic methods are much more expensive and yet not completely interference-free (*38*). Haemolysis and hyperbilirubinemia can significantly affect the reliability of enzymatic determination of creatinine, with notable between-method variability in terms of the nature and intensity of the specific interference. In addition, significant interferences of numerous drugs, as well as monoclonal proteins, have been demonstrated in various enzymatic creatinine methods (*39*-*42*).

A good knowledge of both biological and analytical variability factors in creatinine measurement is required, as these have a combined effect on the overall variability of not only creatinine results but also the GFR estimations with predictive equations derived from serum creatinine values (*32*). Namely, due to the hyperbolic relationship between creatinine and eGFR in these equations, even small differences in low creatinine concentration translate into large differences in eGFR. Consequently, misclassification of the degree of renal function may occur. One of the ways to overcome the limitations of creatinine in an ambiguous clinical situation is to use another established marker of glomerular filtration-cystatin C.

## Cystatin C

Cystatin C (previously termed gamma-trace protein) is a 13 kDa protein with cysteine proteinase inhibiting properties, produced by all nucleated cells at a constant rate (*43*). In the kidney, cystatin C undergoes almost complete reabsorption and catabolism in proximal tubules, after being freely filtered through the glomeruli. Considering the renal handling and relative insensitivity to the variables of gender, age, body mass index (BMI) and nutrition habits, serum cystatin C was proposed as an alternative marker of glomerular filtration almost 40 years ago (*44*). Ever since serum cystatin C has been the subject of extensive research in various clinical studies aiming to evaluate its performance in the early detection of CKD (*45*).

The major obstacle in obtaining reproducible and comparable data was a lack of standardization of analytical methods used for the laboratory testing of cystatin C. Global standardization efforts, led by the International Federation of Clinical Chemistry and Laboratory Medicine (IFCC) Working Group on Standardization of Cystatin C resulted in the release of the primary certified cystatin C reference material (ERM-DA471/IFCC) in 2010 (*46*). In the subsequent period reagent manufacturers recalibrated their assays to the IFCC reference standard aiming to achieve better between-method harmonization. However, unacceptable biases and poor compliance to the criteria of acceptable performance based on the biological variability criteria remained unresolved (*47*).

While there is still a need for improvement in serum cystatin C testing, there are also important biological and clinical confounders that limit its use, such as obesity, thyroid dysfunction, systemic inflammation and treatment with corticosteroids (*48*). In addition, routine testing of cystatin C is associated with substantial costs in comparison to serum creatinine, which significantly limits its availability. Nevertheless, the use of serum cystatin C for the glomerular filtration assessment has been endorsed in clinical guidelines, particularly for the confirmation of CKD in the cases with serum creatinine-based eGFR values between 45 and 60 mL/min/1.73 m^2^ in the absence of other markers of kidney damage (*29*). Also, based on the ample evidence of the independent association with mortality and cardiovascular disease in populations with and without CKD, the utility of serum cystatin C in identifying high-risk patients with CKD has been strongly advocated, beyond its use as a glomerular filtration biomarker (*48*-*50*).

There is conflicting evidence on the predictive value of serum cystatin C on diabetes risk. While it was positively linked to increased prevalence of diabetes in middle-aged and older adults, Magnusson *et al.* found an association of serum cystatin C concentrations with metabolic syndrome, but not with type 2 diabetes (*51*, *52*). Nevertheless, the causal associations with adverse outcomes could not be demonstrated in genome-wide association studies, indicating that elevated risks are primarily due to the renal damage that is reflected by elevated serum cystatin C concentrations (*52*, *53*).

Evidence from meta-analyses confirmed an excellent diagnostic performance of the serum cystatin C in the diagnosis of DKD, but the advantage of using serum cystatin C over creatinine in the estimates of GFR in diabetes could not be confirmed (*54*-*56*). Rather, the use of the combined serum creatinine-cystatin C equation seems to better address the need for an accurate GFR estimation and risk stratification for CKD progression in diabetic patients (*57*).

## Glomerular filtration rate assessment

Assessment of GFR is essential for the diagnosis and monitoring of CKD. The measurement of GFR is a complex procedure involving the administration of an exogenous substance (inulin, iohexol or some radionuclide), collection of multiple samples over a defined period, and specific analytical quantitation of the applied marker (*58*). Measurement of GFR is recommended in certain clinical conditions, such as dosing potentially nephrotoxic drugs in oncology patients, evaluating kidney function of a living kidney donor or in patients with extremely high or low BMI (*59*).

Not all methods/markers for the measurement of GFR perform with sufficient accuracy. As evidenced by Soveri *et al.* in a systematic review, whenever a gold-standard renal inulin clearance is not available, sufficiently accurate methods to measure GFR should be renal and plasma clearance of 51Cr-EDTA, renal clearance of iothalamate and plasma clearance of iohexol (*60*). However, it should be noted that the iohexol method due to its simplicity, affordability and a large body of scientific evidence, gained almost universal acceptance and became the method of choice for GFR measurement in the European countries (*61*, *62*).

Endogenous creatinine clearance had been used as a marker of GFR for many years, despite serious drawbacks. Measurement of creatinine clearance required a cumbersome 24h urine specimen collection, which was burdened with a large proportion of collection-associated errors. Moreover, creatinine clearance does not accurately reflect kidney function due to variable and substantial tubular secretion, influenced by numerous biological and pharmacological factors (*31*). Endogenous creatinine clearance consistently overestimated GFR, with relatively higher overestimation at low GFRs (*60*). Consequently, the measurement of creatinine clearance is no longer used as an estimate of GFR.

The need for a practical surrogate marker with sufficient accuracy to assess kidney function led to the development of various predictive equations for the GFR estimation based on serum creatinine values. The first clinically widely used equation able to estimate the creatinine clearance from serum creatinine concentration, adjusted for age and weight, was designed by Cockroft and Gault in 1976 (*63*). It was derived and validated in a small population of hospitalised male patients using unstandardized serum creatinine method results, and suffered from an inherent weakness resulting in significant over- and underestimations in overweight/obese and lean individuals, respectively (*64*). It is no longer recommended for use because it has not been recalibrated for IDMS standardized creatinine methods. Despite limitations and imperfections, the Cockroft-Gault equation provided a practical tool for the assessment of kidney function in various clinical settings and paved the way for the development and validation of other predictive equations with improved performance.

Modification of Diet in Renal Disease (MDRD) Study, conducted in a large cohort of patients with CKD, among other objectives aimed to develop a predictive equation for eGFR from serum creatinine, with ^125^I-iothalamate as a reference method for measured GFR (*65*). Age, gender, plasma creatinine and race (white or black) were found to be the most prominent predictive variables of GFR and were therefore included in a 4-variable MDRD-equation. The equation was re-expressed after the standardization of creatinine measurement by calibration traceable to the IDMS reference method (*66*). Due to the inaccuracy at higher GFR values, a cut-off for reporting was set at 60 mL/min/1.73m^2^, with a recommendation to report the data above the cut-off to be reported as > 60 mL/min/1.73 m^2^, rather than the respective calculated value.

The Chronic Kidney Disease Epidemiology Collaboration Group (CKD-EPI) in 2009 proposed a new serum creatinine-derived equation with improved accuracy at GFR > 60 mL/min/1.73m^2^ (*28*). The equation design encompassed four different equations (males, females, above and below specific creatinine knot value), and different factors were used for Caucasians and African-Americans, respectively. The original SCr-CKD-EPI equation provided a more accurate estimation of GFR at higher values, which is particularly interesting in diabetic patients with higher eGFR, and was soon assigned as the recommended equation for automated eGFR reporting in the Kidney Disease: Improving Global Outcomes (KDIGO) international, as well as national clinical and laboratory guidelines (*67*-*69*).

At the same time, two additional equations, one using standardized cystatin C alone and the other using a combination of cystatin C and standardized creatinine, were developed by the CKD-EPI Group, seeking to improve accuracy and overcome the limitations of the SCr-CKD-EPI-based estimates of GFR (*70*). The validation study demonstrated that the combination of serum creatinine and serum cystatin C was more accurate than either marker alone for estimating GFR in various patient populations. However, a recent meta-analysis could not demonstrate greater accuracy of serum cystatin C-based equations, either alone or combined with serum creatinine, in comparison to measured GFR in patients with diabetes, probably due to high variability of the cystatin C assays due to an incomplete standardization (*56*).

Today serum cystatin C is gaining an increasingly prominent place in the screening for CKD, particularly regarding the 2021 CKD-EPI eGFR equations without race (*71*). Namely, the adoption of the SCr-CKD-EPI equation and the practice of automatically including the respective eGFR in the laboratory report whenever serum creatinine is tested, enabled a wide screening for CKD but was also surrounded by controversies regarding the variable of race that was included in the equation. Apart from race being a social, rather than biological entity, the use of the 2009-CKD-EPI equation not only may have contributed to medical racism but also a miss-classification of CKD with inadequate use of race-specific equations. The 2021-CKD-EPI equations contain only variables of age, sex and either serum creatinine or cystatin C alone, or a combination of serum creatinine and cystatin C. While the new SCr-eGFR equation without race (Table 2) was found to have sufficient accuracy for clinical practice in many circumstances, the combined SCr-Cys-eGFR equation without race (Table 3) showed a better concordance to the measured GFR and a smaller bias between race groups. The 2021-CKD-EPI equation was immediately recognized by medical laboratories as an opportunity for further standardization of GFR estimation and reporting, and practical guidance was issued aiming to facilitate the transition throughout the laboratory community in the USA (*72*).

**Table 2:** 2021 CKD-EPI equations to calculate eGFR from serum creatinine

**Age (years)**	**Sex**	**SCr (µmol/L)**	**eGFR equation**
≥ 18	Female	≤ 61.9	142 x (SCr / 61.88)^-0.241^ x 0.9938^Age^ x 1.012
		> 61.9	142 x (SCr / 61.88)^-1.200^ x 0.9938^Age^ x 1.012
	Male	≤ 79.6	142 x (SCr / 79.56)^-0.302^ x 0.9938^Age^
		> 79.6	142 x (SCr / 79.56)^-1.200^ x 0.9938^Age^
SCr - serum creatinine. eGFR - estimated glomerular filtration rate. Adapted from reference 67.			

**Table 3:** Equations to calculate eGFR from serum creatinine and cystatin C

**Age (years)**	**Sex**	**SCr (µmol/L)**	**SCys (mg/L)**		**eGFR_Cr-Cys_**
≥ 18	Female	≤ 61.9	≤ 0.80	135 x (SCr / 61.88)^-0.219^ x (SCys / 0.8)^-0.323^ x 0.9961^Age^ x 0.963	
			> 0.80	135 x (SCr / 61.88)^-0.219^ x (SCys / 0.8)^-0.778^ x 0.9961^Age^ x 0.963	
		> 61.9	≤ 0.80	135 x (SCr / 61.88)^-0.544^ x (SCys / 0.8)^-0.323^ x 0.9961^Age^ x 0.963	
			> 0.80	135 x (SCr / 61.88)^-0.544^ x (SCys / 0.8)^-0.778^ x 0.9961^Age^ x 0.963	
	Male	≤ 79.6	≤ 0.80	135 x (SCr / 79.56)^-0.144^ x (SCys / 0.8)^-0.323^ x 0.9961^Age^	
			> 0.80	135 x (SCr / 79.56)^-0.144^ x (SCys / 0.8)^-0.778^ x 0.9961^Age^	
		> 79.6	≤ 0.80	135 x (SCr / 79.56)^-0.544^ x (SCys / 0.8)^-0.323^ x 0.9961^Age^	
			> 0.80	135 x (SCr / 79.56)^-0.544^ x (SCys / 0.8)^-0.778^ x 0.9961^Age^	
SCr - serum creatinine. SCys - serum cystatin C. eGFR - estimated glomerular filtration rate. Adapted from reference 67.					

However, the European perspective regarding the implementation of the 2021-CKD-EPI equation is not affirmative due to very recent evidence. Fu *et al.* have shown that the implementation of the 2021-CKD-EPI equation, yielding slightly higher eGFR than the 2009-CKD-EPI equation, resulted in a shift of the significant proportion of CKD patients to a higher GFR category, with substantial epidemiological and clinical consequences, ranging from a decrease of the estimated CKD prevalence to a delay of appropriate medical management in the white European population (*73*). Furthermore, the new equation performed with an inferior accuracy in European white and European black, as well as in African black populations (*74*). It was therefore concluded by Gansevoort *et al.* from the European Renal Association (ERA) that the adoption of the new equation offers no improvement over the currently used 2009-CKD-EPI equation for the white population, and the change of practice be left for the better-performing equation after the scrupulous validation and a reach of global consensus (*75*). At the same time, a position statement by the Task group Chronic Kidney Diseases of the European Federation of Clinical Chemistry and Laboratory Medicine (EFLM) recommended not to implement the 2021-CKD-EPI equation and to continue using the 2009-CKD-EPI equation without correction for the race (*76*).

The European Kidney Function Consortium (EKFC) developed a new type of eGFR equation, which uses sex- and age-specific median creatinine values in healthy subjects (“Q-values”) for GFR estimation of individual subjects in the full age spectrum (*77*). Very recently, an EKFC eGFR-Cystatin C-based race- and sex-free equation was published, applying the same mathematical principle as EKFC creatinine-based equation and performing with a superior accuracy over routinely used equations in cohorts from Europe, USA and Africa (*78*).

The diagnostic performance of the new equations is yet to be determined in the specific subgroups of patients with diabetes. Current clinical guidelines recommend at least annual eGFR assessment in patients with type 1 diabetes with duration of years and in all patients with type 2 diabetes regardless of treatment, and 1-4 times *per* year monitoring in patients with an established CKD (*26*).

## Urinary albumin

Elevated urinary albumin excretion, also termed albuminuria, is another essential test for the screening and management of chronic kidney disease, particularly in diabetes.

Small amounts of albumin (< 30 mg/day) are normally filtered through glomeruli and excreted in the urine. Albuminuria, resulting from pathological aberrations in the selective permeability of the glomerular basement membrane, is not only an early sign of DKD but also an independent risk factor for cardiovascular morbidity and mortality even in the absence of diabetes (*19*). Current clinical guidelines have defined three categories of albuminuria with fixed cut-off values that are used to assess the stage and monitor the progression of CKD (Table 1). Similarly to other diagnostic biomarkers, the use of fixed clinical decision-making thresholds requires standardized laboratory methodology yielding comparable results to avoid misclassification and adverse patient outcomes. In the case of albuminuria, neither analytical methods nor the type of sample and the reporting units were standardized, which introduced a substantial amount of uncertainty in the clinical decision-making processes related to CKD (*79*). Albuminuria testing can be carried out in both 24h urine, timed urine, and spot urine samples, and the results are reported as either urinary albumin excretion rate (mg/24h, mg/min) or albumin/creatinine ratio (mg/g or mg/mmol), respectively.

The National Kidney Disease Education Program Laboratory Working Group (NKDEP-LWG) together with the International Federation of Clinical Chemistry and Laboratory Medicine Working Group for Standardization of Albumin in Urine (IFCC WG-SAU) started a joint standardization programme for urinary albumin in 2008, after the major flaws in albuminuria testing had been identified (*80*). The standardization program has been operative ever since with the main goals: 1) to establish a reference procedure and reference materials for the measurement of albumin in urine, and 2) to address the reporting issues, primarily for the urine albumin/creatinine ratio to improve harmonization and standardization of the test. So far the program provided two candidate reference measurement procedures and made a substantial effort in providing the primary reference material for urinary albumin (*81*, *82*). Namely, commercial methods for albuminuria testing are calibrated against a certified reference material for serum proteins, including albumin that must be diluted to obtain an appropriate concentration range for urine albumin. Considering variable reagent and dilution-buffer formulations in commercial albuminuria assays, dilution of reference material may introduce a substantial bias. This was evidenced by a comparison study of 17 commercially available urinary albumin methods to a candidate reference method, where bias was revealed as the dominant source of discrepancies among the methods (*83*). The National Institute of Standards and Technology (NIST) is developing two candidate standard reference materials (SRMs): 2925 Pure Albumin, released in 2020 and intended for calibration of the primary reference methods, and 3666 Albumin in Frozen Human Urine, intended for use as a four-level calibration material for commercially available methods including urinary creatinine as well, with the release date not announced yet (*84*, *85*).

Optimal measurement procedure performance goals for urine albumin were proposed according to the biological variation model as a total allowable error ≤ 24%, bias to a reference measurement procedure ≤ 7% and imprecision ≤ 6% (*85*). These specifications offer a valuable tool for laboratories to evaluate and select a method routinely used for albuminuria testing.

As regards the type of sample and the reporting units, current recommendations endorse the reporting of the albumin to creatinine ratio (ACR), preferably in the first-morning urine sample. A random urine sample may also be used, but the positive ACR results should be confirmed by retesting in the first-morning sample. If a more accurate estimate of albuminuria is required, 24h urine samples should be collected and classification by using appropriate thresholds carried out (Table 1). The recommendation to use ACR rather than the albuminuria quantification in the 24h or timed urine specimens was issued due to obvious practical reasons, but also due to the evidence of the lower biological variability of the former (*80*).

The most recent American Diabetes Association guidelines recommend annual screening for albuminuria by performing ACR in spot urine samples for patients with type 1 diabetes with a duration of > 5 years and for all patients with type 2 diabetes. In patients with an established CKD, ACR should be monitored 1-4 times *per* year, depending on the CKD stage (*26*). Considering a high biological variability (> 20%) in urinary albumin excretion, it is required that two of three ACR results within a 3 to 6-month period be positive to confirm the diagnosis of elevated albuminuria. In addition, when interpreting ACR results it should be recognized that albuminuria may be elevated by numerous non-renal conditions, such as heavy exercise, menstruation, fever, congestive heart disease and unregulated hypertension (*86*).

While the efforts within the standardization program enabled substantial progress in the harmonization of albuminuria testing, clinicians should be aware of the still existing between-method variability due to a lack of traceability to the primary reference standard. Analytical flaws, together with a high biological variability have a considerable impact on the clinical decision-making processes based on albuminuria testing. Thus, there is still a great need for improvement in this essential laboratory test for the diagnosis and staging of DKD.

## Phenotypes of DKD and emerging biomarkers

The classical clinical trajectory of DKD includes 5 stages that progress slowly over time. Stage 1 is characterized by hyperfiltration and normoalbuminuria, thereafter following stage 2 with intermittently elevated albuminuria and normal blood pressure. Stage 3 is the initial clinical stage, presenting with persistently elevated albuminuria (A2), mild hypertension, and a normal or moderately decreased GFR. In stage 4, there is severely increased albuminuria (A3), hypertension, and further GFR reduction. The final stage 5 is end-stage renal disease (*5*). However, several non-classical phenotypes of DKD have been recognized as well.

First, there is a substantial proportion of patients, particularly with type 2 diabetes, who progress to CKD with severe GFR decline without or with only slightly elevated albuminuria (*87*). Type 2 diabetic patients with elevated albuminuria seem to follow a typical pattern of diabetic microvascular complication, while those with normal albuminuria and reduced GFR were at higher risk for cardiovascular and macrovascular complications (*88*). Second, some patients develop a rapid decline in kidney function (reduction of eGFR > 5 mL/min/1.73 m^2^/year), which is associated with an increased risk for ESRD, but the mechanism(s) behind this phenotype is still unknown (*89*). Finally, there are patients who experience a regression of albuminuria due to the favourable response to interventions: rigorous control of blood pressure and glycaemia, dietary sodium restriction and renoprotective treatment with SGLT2 inhibitors (*90*). The pathways eliciting glomerular recovery and conversion to normoalbuminuria have not been elucidated.

Although some clinical risk factors such as age, diabetes duration, HbA1c, systolic blood pressure, and retinopathy have been associated with various phenotypes, the addition of clinical factors to albuminuria and eGFR only modestly improved the prediction of GFR decline (*91*). Therefore, additional predictive biomarkers are needed to enable early differentiation and guide interventions across the spectrum of DKD phenotypes.

In the past decades numerous urinary and plasma biomarkers reflecting the pathophysiology of DKD have been investigated, but none has reached the performance for diagnostic use so far. These have been extensively reviewed elsewhere (*5*, *12*, *91*-*93*). For the purpose of this review, it can briefly be summarized that among the various biomarkers of inflammation, endothelial dysfunction, fibrosis and tubular damage that showed promise in the initial studies (Table 4), only a few of them were independently associated with renal outcomes in large follow-up studies. Among the single biomarkers of inflammation, elevated concentrations of the soluble TNF-alpha receptors 1 and 2 were identified in multiple studies and various populations as strong predictors of fast renal decline to ESRD in both type 1 and type 2 diabetes (*89*). Kidney injury molecule-1 (KIM-1), a proximal tubular apical membrane protein is so far the most researched tubular marker in DKD. Plasma and urinary concentrations of KIM-1 increase with albuminuria and eGFR decline, and a causal link between KIM-1 and kidney function were suggested by Mendelian randomisation, based on genome-wide association study data for the KIM-1 gene (*94*). However, the predictive value of KIM-1 for the risk of CKD progression could not be demonstrated in longitudinal studies, neither in type 1 nor in type 2 diabetic patients (*94*, *95*).

**Table 4:** Candidate biomarkers associated to diabetes kidney disease

**Renal biomarkers**	
Glomerular	α_1_-Microglobulin β_2_-Microglobulin
Tubular	KIM-1 NGAL L-FABP FGF23 Osteopontin Uromodulin Copeptin
**Extrarenal biomarkers**	
Inflammation/endothelial dysfunction	TNFR1/TNFR2 ADM FGF21 VEGF TGFβ-1 SDMA/ADMA MMPs Fetuin A
“Omics”-derived multimarkers	CKD273
KIM-1 - kidney injury molecule-1. NGAL - neutrophil gelatinase-associated lipocalin. L-FABP - liver-type fatty acid-binding protein. FGF23 - fibroblast growth factor 23. TNFR1/TNFR2 - tumour necrosis factor receptor 1/tumour necrosis factor receptor 2. ADM - adrenomedullin. FGF21 - fibroblast growth factor 21. VEGF - vascular endothelial growth factor. TGFβ-1 - transforming growth factor beta 1. SDMA/ADMA - symmetric dimethylarginine/asymmetric dimethylarginine. MMPs - Matrix metalloproteinases. CKD273 - chronic kidney disease classifier 273.	

Apart from single biomarkers, a multimarker approach using bioinformatics modelling based on a literature data search also provided evidence of the value of several sets of biomarkers for prediction of the progression of DKD (*96*, *97*). Finally, the “omics” technologies, which enabled a global search for the novel biomarkers throughout the entire human biology and paved the way to personalized medicine, found a prominent place in the DKD studies as well. So far, the urinary peptides-based chronic kidney disease classifier 273 (CKD273) derived from proteomic studies has been reported as an independent predictor of microalbuminuria in normoalbuminuric type 2 diabetic patients (*98*). In a recent analysis of the urinary peptides from the human urine proteome database, the association of peptides derived from fetuin-A with impaired kidney function in T2DM patients was found (*99*). In addition, metabolomics studies revealed several metabolites associated with early changes in DKD, whereas genomic and transcriptomic data are expected to provide a more precise insight into the pathogenesis of DKD in the future (*100*-*102*).

While it is beyond doubt that “omics” approaches have a great potential for identification of new biomarkers, many scientific and technological efforts are required for the diagnostic validation, standardization and the development of analytical and post-analytical tools to achieve applicability in routine clinical practice.

## Conclusion

Early detection and prevention of diabetes complications, including diabetic kidney disease, is one of the major challenges of modern medicine. Although a decline in the incidence of CKD and improved outcomes in patients with DKD have been achieved because of diagnostic and therapeutic advances, shortcomings still exist.

While various renoprotective strategies are being investigated in the field of interventions, the identification of risk phenotypes and the detection of early changes in the kidney that can serve as a target for specific interventions are of crucial importance on the diagnostic side.

Long-established biomarkers such as GFR and albuminuria will certainly continue to be the cornerstone of diagnosis and risk stratification in routine clinical practice. However, their immanent biological limitations and analytical variations discussed in this review should be rigorously addressed and taken into account in the clinical interpretation of the results. Continuous improvements in predictive equations, standardization/harmonization of the recommended laboratory tests and a wider availability of cystatin C testing shall contribute significantly to the quality of patient care.

Considering the complex and diverse pathophysiology of DKD, especially in type 2 diabetes, a panel of biomarkers would be needed to enable the classification of patients in terms of the rate of disease progression and/or response to specific interventions. Personalized approach to the diagnosis and treatment of DKD in the future, will facilitate a better response to this challenging disease and enable improved outcomes for a large number of patients worldwide.

## Data Availability

No data was generated during this study.
